# A systematic review of the use of health services by immigrants and native populations

**DOI:** 10.1186/s40985-016-0042-3

**Published:** 2016-12-03

**Authors:** Antonio Sarría-Santamera, Ana Isabel Hijas-Gómez, Rocío Carmona, Luís Andrés Gimeno-Feliú

**Affiliations:** 1 Red de Investigación en Servicios de Salud y Enfermedades Crónicas, Madrid, Spain; 2 National School of Public Health, Institute of Health Carlos III, Madrid, Spain; 3 IMIENS, UNED, Madrid, Spain; 4 Faculty of Medicine, University of Alcalá, Alcalá, Spain; 5 University Hospital Fundación Alcorcón, Alcorcón, Spain; 6 Agency for Health Technology Assessment, Institute of Health Carlos III, Madrid, Spain; 7 EpiChron Research Group on Chronic Diseases, Aragon Health Sciences Institute (IACS), IIS Aragón, Zaragoza, Spain; 8 San Pablo Health Centre, Aragonese Health Service, Zaragoza, Spain; 9 Department of Medicine, Psychiatry and Dermatology, University of Zaragoza, Zaragoza, Spain

**Keywords:** Access to health care, Immigrants and native born

## Abstract

**Background:**

Changes in migration patterns that have occurred in recent decades, both quantitative, with an increase in the number of immigrants, and qualitative, due to different causes of migration (work, family reunification, asylum seekers and refugees) require constant u pdating of the analysis of how immigrants access health services. Understanding of the existence of changes in use patterns is necessary to adapt health services to the new socio-demographic reality. The aim of this study is to describe the scientific evidence that assess the differences in the use of health services between immigrant and native populations.

**Methods:**

A systematic review of the electronic database MEDLINE (PubMed) was conducted with a search of studies published between June 2013 and February 2016 that addressed the use of health services and compared immigrants with native populations. MeSH terms and key words comprised Health Services Needs and Demands/Accessibility/Disparities/Emigrants and Immigrants/Native/Ethnic Groups. The electronic search was supplemented by a manual search of grey literature. The following information was extracted from each publication: context of the study (place and year), characteristics of the included population (definition of immigrants and their sub-groups), methodological domains (design of the study, source of information, statistical analysis, variables of health care use assessed, measures of need, socio-economic indicators) and main results.

**Results:**

Thirty-six publications were included, 28 from Europe and 8 from other countries. Twenty-four papers analysed the use of primary care, 17 the use of specialist services (including hospitalizations or emergency care), 18 considered several levels of care and 11 assessed mental health services. The characteristics of immigrants included country of origin, legal status, reasons for migration, length of stay, different generations and socio-demographic variables and need. In general, use of health services by the immigrants was less than or equal to the native population, although some differences between immigrants were also identified.

**Conclusions:**

This review has identified that immigrants show a general tendency towards a lower use of health services than native populations and that there are significant differences within immigrant sub-groups in terms of their patterns of utilization. Further studies should include information categorizing and evaluating the diversity within the immigrant population.

## Background

The number of international migrants continues to grow each year. According to the United Nations Migration Report, the number of migrants has reached 244 million in 2015 up from 191 million in 2005, representing an increase of 28% over the decade in comparison with an increase of 13% during the period 1990–2000 [1, 2].

Between 2000 and 2015, Europe has absorbed the second largest number of international migrants following Asia [1, 3]. Despite the global economic crisis which started in 2007–2008, Europe and Northern America have recorded an annual growth rate in the international migrant stock of 2% per year [1].

These transformations have both quantitative (i.e. an increasing number of migrants) and qualitative (i.e. evolving reasons for migration) aspects. There is a trend towards permanent migration and reunification of families with immigrant setting in the host country in a more definitive way [4]. And most recently, we have seen an increasing number of asylum seekers and refugees, which is reaching the highest levels seen since World War II [1].

This situation has generated various responses in the host countries, as immigration is acquiring a significant social and political dimension. Immigration is influencing public opinion and triggering a debate, often improperly informed, regarding the pressure on public services—including health services [3]. This has even led to the adoption of new legislation [5–7] limiting access to health care for migrants, that may pose, as a result, a risk to public health.

The dramatic changes in demographics, socio-economics and politics require an update of the analysis of health service utilization by immigrants in order to properly determine the breadth and scope of the current situation. Consequently, research on migrant access and utilization of health services has proliferated in recent decades [8, 9]. Results from a previous review point to a lower utilization rate of general and specialist medical services by immigrants compared to native-born populations [10]. However, and since patterns of healthcare utilization depend on factors that may have evolved in recent years, such as age, sex, socio-economic level, time of stay in the host country or origin of the immigrants, and the specific features of healthcare services of the host countries, it seems necessary to revisit the state of knowledge on this subject.

The objective of this study is to describe the available scientific evidence that has investigated the differences in healthcare service utilization between immigrant and native populations in the last 3 years (June 2013 through February 2016), and to explore the possible effect on the differential use of variables associated with health needs, socio-economic status or other factors.

## Methods

A systematic literature review was performed to identity the available empirical evidence comparing immigrant’s healthcare utilization with native populations using a predefined protocol [10]. Inclusion criteria for articles to be considered were original studies with quantitative data that compared the use of healthcare services between native and immigrant populations. Service use was defined as the interaction between health professionals and patients [11]. Only studies with both population groups properly defined, i.e. immigrant and native, were included. For the purposes of this review, we used the European Union definition of immigrant status based on foreign country of birth including up to the second generation [12].

Papers that considered undocumented immigrants, asylum seekers and/or refugees were also included. The indigenous majority population served as the native reference group. No limitation in gender or ethnic characteristics was stipulated.

Articles were excluded if they (1) exclusively evaluated healthcare utilization for children or adolescents younger than 18 years of age, (2) were editorials, letters or reviews and (3) were qualitative studies.

### Search strategy and study selection

Two strategies were utilized in the search for relevant articles on this review.

Firstly, in February 2016, a librarian conducted a systematic review of the electronic database MEDLINE (PubMed) in search of the literature published between June 2013 and February 2016. No language restrictions were applied; no authors were contacted for additional information. MeSH terms and key words used, as well as search strategies performed, are shown in Table 1.

**Table 1:** Search strategy for healthcare service utilization’s comparative studies

General practitioner use (electronic search):		
1. Health Services Needs and Demand/	12. health services [Title]	23. 18–22 / OR
2. Health Status/	13. Primary care [Title]	24. immigrant* [Title]
3. Health Services Accessibility/	14. Emergency services [Title]	25. migrant* [Title]
4. Coverage [Title]	15. Utilization patterns [Title]	26. Ethnic Groups [Title]
5. 1–4 / OR	16. 6–15/ OR	27. 24–26 / OR
6. health care [Title]	17. 5 and 16	28. 23 and 27
7. health disparities [Title]	18. Emigration and Immigration/	29. Health AND utilization AND immigrant* [Title]
8. access to care [Title]	19. Emigrants and Immigrants/	30. 17 AND 28
9. health resources [Title]	20. Native [Title]	31. 29 or 30 (GPs precise search)
10. health profiles [Title]	21. Foreign [Title]	32. (16 AND 27) OR 29 (GPs exhaustive search)
11. health status [Title]	22. Autochthonous [Title]	
Specialist use (electronic search):		
1. Health Services/utilization/	7. Emigrants and Immigrants/	13. Specialization/
2. Health Services Accessibility/	8. Ethnic Groups	14. speciali* [TI]
3. Health Status/	9. Native [Title]	15. 13 OR 14
4. Coverage [Title]	10. Foreign [Title]	16. 5 AND 12 AND 15
5. 1–4 / OR	11. Autochthonous [Title]	
6. Emigration and Immigration/	12. 6–11 / OR	

The initial screening of the articles was based on abstracts. Two researchers reviewed all abstracts independently. Selection of relevant articles was based on the information obtained from the abstracts and was agreed upon in discussion. If the abstract was not available, the full text was examined. In the case of discrepancies between the two researchers, the original paper was obtained and an agreement was achieved after it was read.

Secondly, a researcher (AIHG) conducted a manual search of grey literature through Google Scholar, including published papers from 2013 through February 2016 taking into account the terms (Health care use; Comparison; Immigrants; Natives) and (Needs, demands and barriers; Coverage; Primary care; Emergency services; Utilization patterns; Native; Foreign; Autochthonous; Immigrant). Both English and Spanish web pages were included in the search results. Appropriateness for inclusion was based on titles; in the event of doubt, abstracts were retrieved. Studies without electronic abstracts were not included.

Subsequently, two researchers examined the full text of all papers that satisfied the inclusion criteria (AIHG, ASS).

### Data extraction

The following information were extracted from each publication: context of the study (country and year), characteristics of the included population (definition of native and immigrants groups, sample size for each group), methodological components (design of the study, statistical analysis, source of information), area of healthcare services assessed, confounders affecting healthcare utilization (individual determinants, measures of need, socio-economic indicators, cultural factors), objective of the study and main results.

## Results

### Characteristics of the studies

**Fig. 1:**
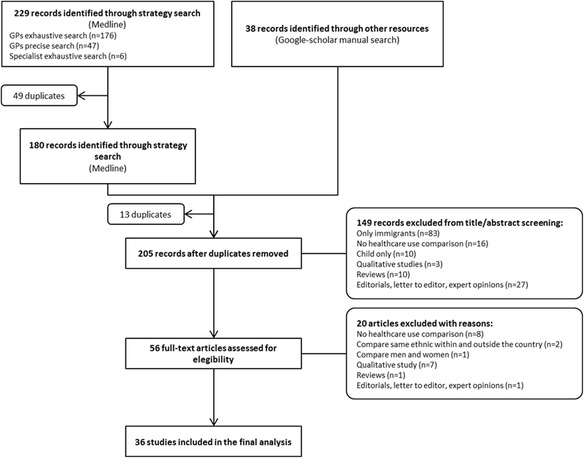
Study flowchart for the selection process of the final included studies

Thirty-six papers met the inclusion criteria in this study. The process followed to include those papers is shown in Fig. 1. Table 2 shows the information extracted from the included publications. Of the 36 studies included, 8 were duplicated in both the manual and electronic search [13–20], 12 were included after the manual search [21–32] and 16 through the electronic search [33–48]. Among them, at least 9 partly describe the same dataset [13–16, 19, 20, 25, 47, 48]. Nevertheless, as these articles focused on different aspects of healthcare use or outcome measures, all were included in this review.

**Table 2:** Descriptive summary of the studies included in the review

Reference	Country	Year	Sample	Objectives	Information sources	Dependent variable	Independent variable (migrant definition)	Need indicators	Socio-economic indicators	Results
Almeida LM et al. [33] 2014	Portugal	2012	277 women Migrants (*n =* 89) Portuguese (*n =* 188)	To evaluate differences in obstetric care between immigrant and native women in a country with free access to health care	Register and survey-based study (1) Administrative databases of the four public maternity hospitals (February 1 and December 31, 2012) (2) Telephone survey	(1) First appointment at >12 weeks (2) Number of prenatal visits	(1) Native: born in Portugal (2) Immigrant: born outside Portugal with both parents born outside Portugal	Age Parity	Family income Education level Marital status	Migrants were more prone to late prenatal care (first pregnancy appointment after 12 weeks of pregnancy, to have fewer than three prenatal visits)
Beiser M et al. [21] 2014	Canada	2009–2010	98,346 individuals Native born (*n =* 83,949) Established migrants (*n =* 10,810) Recent immigrants (*n =* 3587) 20–74 years	To examine the effects of chronic health conditions, as well as personal resources and regional context on labour force participation, receipt of government transfer payments and use of health services by short- and long-stay immigrants compared with native-born Canadians	Survey-based study Canada Community Health Survey (CCHS)	(1) GP visits in the past 12 months (2) Labour force participation (3) Use of government transfer payments	(1) Native-born Canadians (2) Recent immigrants (resident in Canada for 10 years or less) (3) Established immigrants (present in Canada for more than 10 years)	Age & gender Chronic physical conditions (last 6 months or more) Chronic mental conditions	Education level Marital status Official-language ability (English or French) Geographic region	Recent immigrants healthy or with chronic health problems made fewer GP visits Established immigrants with chronic conditions did not differ in their use of GP
Berchet C [22] 2013	France	2006–08	12,999 individuals French (*n =* 11,934) Immigrants (*n =* 1065) ≥18 years	To highlight factors generating healthcare use inequalities relating to immigration	Survey-based study Health Survey (l’Enquête sur la santé et la protection sociale-ESPS)	(1) GP visits (last year) (2) Specialist medical visits (last year)	Nationality and country of birth (subject and parents)	Age & gender Self-rated health Chronic disease and functional limitations Health behaviour (smoke, overweight)	Health insurance Education level Employment status Family composition Isolation and social support Place of residence GP’s and specialist’s patient load	Immigrants present a lower demand for GP and specialist care
Carmona-Alférez MR [23] 2013	Spain (Madrid)	2006–2007	835,401 individuals Natives (*n =* 694,716) Immigrants (*n =* 140,685) 25–64 years	To evaluate the relationship between birthplace of users of PHC in the Community of Madrid (CM) and the referrals to specialists	Register-based study Medical records of PHC (OMI-AP)	(1) Referral to specialists (2) Number of referrals	Country of birth	Age & gender Health problems (last 12 months) Number of visits to the GP (last 12 months) Territorial per capital income GP’s patient load	–	Immigrants from South America had higher probability to be referred for any health problem, while Asiatic immigrants have the lowest overall probability of referrals Immigrants from Western countries, Central America and the Caribbean showed similar referral rates to Spanish natives
De Back TR et al. [34] 2015	Netherlands	2009–2010	60,852 patients with hypertension, ischemic heart disease, cerebrovascular accidents and cardiac failure Native Dutch (*n =* 55,320) Immigrant Moluccan immigrant (*n =* 5532)	To determine the frequency of visits to the medical specialist and GP and the prescription of cardiovascular agents among Moluccans compared to native Dutch	Register-based study Registry data from the Achmea Health Insurance Company (Achmea)	(1) Number of GP visits (2) Number of specialist (cardiologist and neurologist) visits	Moluccan and Dutch surnames	Age & gender	Socio-economic status (SES) Area-level SES scores were composed by the Netherlands Institute for Social Research Place of residence	Cardiovascular healthcare use of ethnic minority groups may converge towards that of the majority population
De Luca G et al. [24] 2013	Italy	2004–2005	102,857 individuals Natives (*n =* 97,229) Immigrants (*n =* 5628) 0–64 years	To explore differences in utilization of health services between the immigrant and the native-born populations	Survey-based study Italian Health Conditions survey (ISTAT-Condizioni di salute e Ricorso ai Servizi Sanitari)	(1) GP visits (last 4 weeks) (2) Specialist medical visits (last 4 weeks) (3) Phone consultations (last 4 weeks) (4) ED care visits (last 4 weeks)	Country of birth and citizenship criteria (1) Native (Italian citizens born in Italy) (2) First-generation immigrants (individuals born outside of Italy without Italian citizenship) (3) Second-generation immigrants (individuals born in Italy without Italian citizenship) (4) Naturalized Italians (individuals born outside of Italy with Italian citizenship)	Age & gender Self-assessed family wealth Self-assessed health status Chronic diseases and disability conditions Health behaviour (smoke, weight-checking, physical activity)	Education level Marital status Employment status Number of children in the household Area of residence	Immigrants tend to use specialist services and have telephone consultations less frequently, whereas they use ED services more often
Díaz E et al. [13] 2015	Norway	2008	25,915 patients diagnosed with dementia or memory impairment in PHC Natives (*n =* 25,117) Immigrants (*n =* 788) ≥50 years	To study utilization of primary healthcare services of Norwegians and immigrants with either a diagnosis of dementia or memory impairment	Register-based study (1) National Population Register-NPR (2) Norwegian Health Economics Administration database-HELFO (3) Norwegian Prescription Database-NorPD	(1) Number of GP visits (2) ED visits (3) Home consultations	Country of birth. (Born abroad with both parents from abroad)	Age & gender	Education level Marital status Length of stay in Norway Place of residence	No differences in the use of PHC were found
Díaz E et al. [14] 2014	Norway	2008	3,739,244 individuals Natives (*n =* 3,349,721) Immigrants (*n =* 389,523) ≥15 years	To describe and compare the use and frequency of use of PHC services between immigrants and natives in Norway To investigate the importance of morbidity burden, socio-economic status and length of stay in Norway for immigrants’ use of PHC services	Register-based study (1) National Population Register (2) Norwegian Health Economics Administration database-HELFO	(1) Percentage of each population who had used the PHC system (GPs, EPC and both) in 2008 (2) Frequency of use among PHC users	Country of birth (1) Natives (born in Norway with both parents born in Norway) (2) Immigrants (born abroad with both parents from abroad) staying at least 6 months, divided according to the World Bank income categories of their country of origin	Age & gender Morbidity groups (Johns Hopkins University Adjusted Clinical Groups)	Education level Marital status Income level Place of residence	Significantly fewer immigrants from all but LIC used their GP and all PHC services, but a higher share of immigrants except those from HIC used the EPC. This higher use did not compensate for less use of GPs in terms of overall use of PHC Among GP users, however, immigrants used the GP at a statistically significant higher rate compared with natives Immigrants 65 years from all but HIC used GPs less than other age groups, and the same was true for overall use of PHC, although older immigrants from LIC used the EPC most The use of PHC services, but not the rate of use, increased with length of stay in Norway
Díaz E et al. [15] 2014	Norway	2008	1,605,873 individuals Natives (*n =* 1,516,012) Immigrants (*n =* 89,861) ≥50 years	To describe the utilization of PHC in Norway in terms of number of consultations, diagnoses given and procedures undertaken To compare native Norwegians’ use of PHC services with that of different immigrant groups	Register-based study (1) National Population Register (2) Norwegian Health Economics Administration database-HELFO	(1) Frequency of use of PHC system (GP, EPC) in 2008 (2) Diagnoses received at GP and EPC consultations	Country of birth (1) Natives (born in Norway with both parents born in Norway) (2) Immigrants (born abroad with both parents from abroad) staying at least 6 months, divided according to the World Bank income categories of their country of origin	Age & gender Morbidity groups (Johns Hopkins University Adjusted Clinical Groups)	Education level Marital status Income level Length of stay in Norway Place of residence Reason for migration Age at migration	A lower proportion of HIC immigrants used PHC, but utilization was increasingly similar in older age groups The mean number of consultations to both the GP and the EPC, and the mean number of different diagnoses for PHC users were higher for 50 to 65 years old OIC immigrants, but this pattern was reversed for older adults
Durbin A et al. [25] 2015	Canada (Ontario)	1993–2012	1,820,443 individuals Long-term residents (*n =* 908,329) Immigrants (*n =* 912,114) 18–105 years	Examine the use of primary care and specialty services for non-psychotic mental health disorders by immigrants to Ontario Canada during their first 5 years after arrival	Register-based study (1) OHIP claims data (2) Canadian Institute for Health Information’s Discharge Abstract Database (3) Ontario Mental Health Reporting System (4) National Ambulatory Care Reporting System (April 1, 1993–March 31, 2012)	1) Visits to PHC physicians 2) Visits to psychiatrists 3) Composite of ED visits or hospital admissions	Country of birth (1) Long-term residents (newcomer before 1985 and Canadian-born) (2) Immigrants (identified through the Ontario Citizenship and Immigration Canada (CIC) database)	Age & gender	Education level Marital status Income level Length of stay Official language speaking ability Immigrant admission category Neighbourhood	Immigrants were more or less likely to access primary mental health care depending on the world region of origin Regarding specialty mental health care (psychiatry and hospital care), immigrants used it less. Across the 3 mental health services, estimates of use by immigrant region groups were among the lowest for newcomers from East Asian and Pacific and among the highest for persons from Middle East and North Africa
Durbin A et al. [16] 2014	Canada (Ontario)	2002–2012	359,673 individuals LT-Residents (*n =* 163,263) Immigrants (*n =* 163,298) 18–105 years	To compare service use (primary care visits, visits for psychiatric care, and hospital use) for non-psychotic mental disorders by recent immigrants by matched long-term residents	Register-based study (1) OHIP claims data (2) Canadian Institute for Health Information’s Discharge Abstract Database (3) Ontario Mental Health Reporting System (4) National Ambulatory Care Reporting System	(1) Visits to PHC physicians (2) Visits to psychiatrists (3) Composite of ED visits or hospital admissions	Country of birth (1) Long-term residents (newcomer before 1985 and Canadian-born) (2) Immigrants (identified through the Ontario Citizenship and Immigration Canada (CIC) database)	Age & gender	Education level Income level Official language speaking ability Immigrant admission category Neighbourhood	Immigrants in all admission classes and of both sexes were generally less likely to use all three types of mental health service. The exceptions were for primary mental health care, where male refugees were more likely to have at least one visit For PHC, estimates of intensity of use were highest for refugees and lowest for economic class immigrants For psychiatric care and hospital care, estimates were similar across admission class groups
Esscher A et al. [35] 2014	Sweden	1988–2010	74 individuals Natives (*n =* 48) Immigrants (*n =* 26)	To identify suboptimal factors of maternity care related to maternal death as it occurred in Sweden over a period of increased migration of childbearing women from LIC and MIC	Register-based study (1) Swedish official and national registries (1988–2007) (2) Swedish Society of Obstetrics and Gynaecology (SFOG) Maternal Mortality Group (2008–2010)	Factors of suboptimal care (1) Delay of care-seeking (non-compliance, late booking) (2) Accessibility of services (language proficiency, legal status, transport) (3) Quality of care (Insufficient surveillance and delayed treatment, miscommunication between providers, limited use of resources)	Country of birth divided according to the World Bank Income categories (1) LIC (Ethiopia, Eritrea, Somalia, Democratic Republic of Congo, Zimbabwe, Gambia, and Pakistan) (2) MIC (Poland, Former Yugoslavia, Turkey, Iran, Iraq, Morocco, Philippines, and Thailand)	Age Causes of death	–	Suboptimal care was a significantly more frequent contributing factor of maternal death for the foreign-born women. Many of these deaths were associated with communication-related barriers and delays in care-seeking Immigrant lower health coverage represents the first factor generating inequalities in the propensity to contact a GP, while education and income are the most important drivers of inequalities in the propensity to contact a specialist
Fosse-Edorh S et al. [36] 2014	France	2002–2007	13,959 individuals Born in France (*n =* 12,711) Born in North Africa (*n =* 327) ≥45 years	The objective of the present study was to determine DT2 prevalence and management in immigrants from North Africa living in France to ascertain whether the higher diabetes mortality observed in this population compared with the French-born population reflected a higher prevalence of DT2, poorer health status and or lower quality of care	Survey-based study (1) Population-based survey Enquête décennale santé (EDS; Decennial Health Survey) 2002–2003 (2) ENTRED (Échantillon national témoin représentati des personnes diabétiques; National representative sample of people with diabetes) survey 2007	(1)GP visits last year (2) ≥ 1 private specialist (ophthalmologist or endocrinologist) visit last year (3) Hospitalization >24 h last year 4) Length of stay of hospitalization	Country of birth (1) Born in France (2) Born in North Africa	Age & gender Diabetes complications Smoking	Education level Financial difficulty	Reflects a greater prevalence of DT2, poorer health status and/or lower quality of care in this population Our present study found no major differences between patient groups in terms of medical visits except for less frequent GP and more frequent dentist visits in the BNA population
Franchi C et al. [37] 2016	Italy (Lombardy region)	2010	51,016 individuals Natives (*n =* 25,508) Immigrants (*n =* 25,508) 65–94 years	To compare healthcare resource utilization (drug prescriptions, hospital admissions and healthcare services) in regular immigrants living in the Lombardy Region of Northern Italy at least 10 years versus native elderly people (65 years or older)	Register-based study Administrative databases of Lombardy region (1) Anagraphic database (2) Prescription database (3) Hospital discharge database (4) Outpatient prescriptions by GP (healthcare services utilization)	Drug prescription Polytherapy Hospital admissions Healthcare service utilization	(1) Regular immigrant (born in a country other than Italy and registered with the Italian NHS) (2) Native (born in Lombardy)	Age & gender	–	Older immigrants (65 years and older) present under-utilization of healthcare resources and prescriptions drugs, including those from HIC European countries Only immigrants from Eastern Europe and Eastern Africa have a higher prevalence for hospital admissions. Only immigrants from Northern Africa have higher rate of prescriptions
Garcia-Subirats I et al. [38] 2014	Spain	2006–2007 & 2011–2012	2006–2007 21,818 individuals Natives (*n =* 18,504) Immigrants (*n =* 2893) 2011–2012 15,200 individuals (*n =* 12,559) Immigrants (*n =* 2390) 16–59 years	To analyse the changes in access to health care and the determinants of access among the immigrant and autochthonous populations in Spain between 2006 and 2012	Survey-based study Spanish National Health Survey (SNHS) of 2006–2007 and the SNHS of 2011–2012	(1) Unmet healthcare need in the last 12 months (2) Visits to a GP in the last 4 weeks (3) Visit to a specialist in the last 4 weeks (4) Hospitalization in the last year (5) ED visits in the last year	Country of birth (low and middle-income countries according to the World Bank Income classification)	Age & gender Self-rated health, suffering from a chronic disease, having suffered an injury in the past year	Private health insurance policy Education level Marital status Employment situation Social class (following classification of the Spanish Society of Epidemiology) Length of stay (Immigrants in the SNHS 2011–2012)	In 2012 the immigrant population had a higher prevalence of visiting the GP compared to 2006 The immigrant population had a lower prevalence of visiting the specialist both in 2006 and 2012 The difference in use of ED decreased slightly for both groups and the difference between them was maintained from 2006 to 2012; the immigrant population showed a higher prevalence of use of this care level No significant differences were found between both populations in terms of hospitalizations
Gazard B et al. [26] 2015	United Kingdom, UK (Southeast London, Lambeth and Southwark)	2008–2010	1698 individuals Non-immigrant (*n =* 1010) Immigrants (*n =* 659) ≥16 years	(1) To describe the socio-demographic and socio-economic differences between migrants and non-migrants as broad groupings and by ethnicity, as well as within migrant groups by length of residence in the UK (2) To investigate the associations between migration status and health-related outcomes, including health behaviours, functional limitations, physical and mental health status and health service use (3) To examine whether and how the effect of migration status changes when it is disaggregated by length of residence, first language,reason for migration and combined with ethnicity	Survey-based study South East London Community Health (SELCoH) survey	(1) Registration with GP (2) Visits to a GP for an emotional problem in the last 12 months (3) Seen a counsellor or mental health specialist in the last 12 months (4) Use of hospital services (accident and emergency and other outpatient department) in the last 12 months	(1) Migration status (2) Length of residence in the UK (3) First language (4) Reason for migration (5) Migration status within each ethnic group category	Age & gender Ethnicity	Educational level Employment status Household income Migrant status Length of residence	Migrants who had been in the UK for < 5 years, white migrants and those who migrated for education or work had increased odds of not being currently registered with a GP Migrants who had been in the UK for 5–10 years had increased odds of seeing a GP for an emotional problem. Those who had resided in the UK for <5 years had decreased odds Those who had migrated for education had increased odds of visiting an outpatient department compared to non-migrants decreased odds of seeing a GP for an emotional problem
Gimeno-Feliu LA et al. [27] 2016	Spain (Aragón) & Norway	Norway 2008 & Spain 2010	Native born: Spain (*n =* 1,102,391) Norway (*n =* 4,351,084) Immigrants: Spain (*n =* 35,851) Norway (*n =* 60,733)	Analyse all registered pharmacological treatments for immigrants from Poland, China, Morocco and Colombia compared to natives, aiming to identify patterns of drug use for each immigrant group compared to host countries	Register-based study (1) Pharmaceutical Billing Database in Aragon (2) Norwegian Prescription Database-NorPD	Drug prescription	Country of birth (Poland, Chine, Colombia & Morocco)	Age & gender	–	In the two countries studied, the proportion of immigrants that purchased drugs was significantly lower than that of the correspondingnative population Immigrants from Morocco showed the highest drug purchase rates in relation to natives, especially for antidepressants, pain killers and drugs for peptic ulcer. Immigrants from China and Poland showed lowest purchasing rates, while Colombians where more similar to host countries
Gimeno-Feliu LA et al. [39] 2013	Spain (Aragón)	2007	594,145 individuals Natives (*n =* 527,881) Immigrants (*n =* 66,264) All ages	(1) To analyse the use of primary care services by immigrants compared to Spanish nationals, adjusted by age and sex (2) To analyse the differences in frequency of visits to primary care in relation to geographic origin	Register-based study Electronic medical records register (OMI: Computerized Medical Office)	(1) GP appointments (2) Paediatric appointments (3) Nurse appointments (4) Midwife appointments (5) Physiotherapy appointments (6) Dental appointments (7) Social worker appointments (8) PHC team appointments	Nationality	Age & gender	–	The immigrant population makes less use of PHC services. This is evident for all age groups and regardless of immigrants’ countries of origin
Klaufus L et al. [40] 2014	Netherlands	2008	14,131 individuals Native born (*n =* 11,678) Immigrants (*n =* 2453) >14 years	To investigate ethnic differences as a factor in mental healthcare consumption in patients with medium & high risk of CMD (common mental disorders) and to identify determinants that may explain possible ethnic differences	Survey-based study Health survey conducted by Public Health Services (Amsterdam, Rotterdam, Utrecht and the Hague)	(1) GP visits (last year) (2) Mental health visit (psychiatrist, psychologist or a mental health care facility) last year	Country of birth (subject and parents) (1) Native Dutch (2) First-generation immigrant (foreign born and almost one parent foreign born) (2) Second-generation immigrant (born in Netherland with at least one parent foreign born)	Age & gender Physical health problems	Education level Marital status Employment status Financial situation Social loneliness	Ethnic minority groups contacted the GP significantly more often than native Dutch people, with the exception of Antillean/Aruban immigrants First-generation immigrants tended to contact the GP more often than second-generation immigrants The four ethnic minority groups visited a mental healthcare specialist more often than the Dutch; this was significantly higher among the Turks
Kerkenaar M et al. [41] 2013	Austria	October 2010–September 2011	3448 individuals Natives (*n =* 2930) Immigrants (*n =* 518) ≥15 years	To study: (1) the prevalence of dysphoric disorders among different groups of migrants (first and second generation from different regions) in comparison to the native Austrian population using a validated questionnaire (2) The influence of gender, socio-economic factors, fluency of host language and length of stay in Austria on this prevalence (3) The utilization of healthcare services of migrants and Austrians with and without a dysphoric disorder	Survey-based study (Telephone survey ad hoc and PHQ-4)	(1) Visits to a GP in the last 4 weeks (2) Visits to specialists in their own practices in the last 4 weeks (3) Out or inpatient hospital care in the last 4 weeks (4) Prevalence of dysphoric disorders	Country of birth and country of birth of fathers	Age & gender Chronic disease	Education level Employment status Living area Persons in house	No significant difference was found in the utilization of healthcare services associated with dysphoric disorders, except for a higher utilization of secondary/tertiary care by female migrants with a dysphoric disorder Immigrant males without dysphoric disorders had a lower utilization rate
Koopmans GT et al. [17] 2013	Netherlands	2001–2003	9077 individuals Native Dutch (*n =* 7772) Immigrants (*n =* 1305) ≥18 years	To investigate ethnic-related differences in utilization in outpatient mental health care	Survey-based study Dutch Second National Survey of General Practice (A representative sample of 104 GP practices)	Contact with any mental health service during the last 12 months	Place of birth (subject and parents) Surinamese, Dutch, Antilleans, Moroccans and Turks	Age & gender Self-reported mental health	Education level Marital status Proficiency in Dutch language Orientation towards modern western values Lay views on illness and treatment	Migrant group’s utilization is about half the level of the native Dutch
Lee CH et al. [42] 2013	Singapore	2008–2010	374 patients with diagnosis of STEMI Singapore-born citizens (*n =* 286) Immigrants (*n =* 88)	To study disparities in accessibility to high quality health care, and if patients’ psychosocial condition after discharge was associated with their immigration status	Survey-based study Survey at university-affiliated hospital in Singapore	Patients treated with primary percutaneous coronary intervention, median symptom-to-balloon time, median door-to-balloon time and prescription of evidence-based medical therapy	Place of birth and citizenship (1) Singapore-born citizens (2) Foreign-born citizens (3) Permanent residents	Cardiovascular risk factor profile Admission pathway	Education level Occupation Average monthly household income	There were no major disparities in access to high quality health care for patients with different immigration status
Marchesini G et al. [43] 2014	Italy	2010	7,856,348 patients Italy-born Italian citizens (*n =* 7,328,383) Foreign-born no Italian citizens (*n =* 527,965) All ages	To assess whether prevalence, treatment and direct costs of drug-treated diabetes were similar in migrants and in people of Italian citizenship	Register-based study Administrative data sources of all Italian residents in 30 health districts (ARNO observatory)	(1) Prescriptions (2) Hospitalizations (3) Healthcare services (consultations, laboratory tests and other diagnostic procedures)	Place of birth	Age & gender	Place of residence	Migrants show a higher risk of diabetes but less intense treatment
Pourat N et al. [44] 2014	USA (California)	2009–2010	59,938 individuals Natives (*n =* 8602) Immigrants (*n =* 388) All ages	Test the validity of the assertion that undocumented immigrants are more frequent users of health care	Survey-based study California Health Interview Survey (CHIS)	(1) Number of doctor visits in the past year (2) Percentage of respondents with an ED visits among children and adults in the past year (3) Percentage of children who had a doctor visit in the past year	(1) US-born (2) Naturalized citizen (3) Legal permanent resident or other authorized immigration status (4) Undocumented immigrants	Age & gender Ethnicity Self-assessed health status Number of chronic conditions	Insurance coverage Official Employment status Household income Family status Family size Language (English) proficiency Region of residence Place of residence	Utilization among undocumented immigrants in all analyses was lower than or similar to that of other groups
Ramos JM et al. [28] 2013	Spain (Alicante)	2011	42,839 individuals Natives (*n =* 38,620) Immigrants (*n =* 4219) ≥15 years	To compare hospital admission rates, diagnoses at hospital discharge, service of admission at hospital discharge, and mortality between FCs and autochthonous citizens (ACs)	Register-based study Hospital discharges registries from hospital information systems (Hospital General Universitario de Alicante (HGUA) and Hospital Universitario de Sant Joan d’Alacant (HUS))	Hospital admissions	Foreign citizen (FC) (people without Spanish citizenship) (1) FCs from high income countries (born in 25 European Union countries, Switzerland, Iceland, Norway, the USA, Canada, Japan, and Australia) (2) FCs from low income countries (born elsewhere: North Africa and the Middle East, Latin America, Eastern Europe, Sub-Saharan Africa, and Asia)	Age & gender Diagnosis at discharge Unit of admission Destination at discharge Length of stay	–	The utilization rate was lower in foreign citizens
Rucci P et al. [18] 2015	Italia (Bologna)	2010–2011	8990 individuals Natives (*n =* 8602) Immigrants (*n =* 388) All ages	To determine whether disparities exist in mental healthcare provision to immigrants and natives with severe mental illness	Register-base study Information system of the Departments of Mental Health (DMH), Emilia-Romagna	(1) Receiving psychosocial rehabilitation the following year (2) Days admitted to hospital wards or to residential facilities the following year	Citizenship (immigrants comprise regular immigrants, non-documented immigrants, no Italian citizenship)	Age & gender Mental illness diagnosis Age at first contact Duration of episode	Education level Marital status Working status Living arrangement CMHC area	Although the probability of receiving any mental health intervention is similar between immigrants and Italians, the number of interventions and the duration of admissions are lower for immigrants Immigrants spend less days of residential care in licensed psychiatric facilities or other facilities
Smith-Nielsen S et al. [45] 2015	Denmark	June–August 2007	3,573 individuals Natives (*n =* 1131) Labour immigrants (*n =* 808) RGE immigrants (*n =* 1634) 18–64 years	To investigate whether potential differences exist in the use of private practicing psychiatrists and psychologists	Register and survey-based study Survey and registry study on health and health behaviour of individuals registered at the Danish Civil Registration System (CPR number)	Use of psychiatrist or psychologist last year	Citizenship: (1) Ethnic Danes (at least one parent born in Denmark with Danish citizenship) (2) Immigrant (people residing in Denmark for a minimum of 3 years and born in a foreign country to parents without Danish citizenship) (RGC: Refugee Generating Countries: Turkey, Pakistan, Iraq, Iran, Lebanon, Syria, Somalia and Yugoslavia)	Age & gender Mental health status Physical health symptoms	Marital status Education level Employment status Household income Length of stay in Denmark Oral Danish proficiency	Immigrants from RGC have similar or higher use of psychiatrists and psychologists in private practice when taking mental health into account Labour immigrants in general, except for women using psychiatrists, have lower use of psychiatrists and psychologists
Spinogatti F et al. [29] 2015	Italy	2001–2010	139,775 individuals >17 years	To analyse the differences in mental health service utilization by immigrant and native populations	Register-base study Regional mental health information system Departments of Mental Health (DHM), Lombardy	(1) Contact with psychiatric services (2) Hospitalization in acute psychiatric wards	Country of birth	Age & gender Mental disorder	Marital status Education level Employment status	The treated prevalence of native patients outnumbers that of immigrant ones, although immigrant patients use acute mental health services more frequently
Straiton M et al. [19] 2014	Norway	2008	2,712,974 individuals Natives (*n =* 2,604,757) Immigrants (*n =* 108,217) 18–67 years	To explore treatment options in primary care for immigrant women with mental health problems compared with non-immigrant women	Register-base study National registries (1) National Population Register (2) Norwegian Health Economics Administration database-HELFO (3) Norwegian Prescription Database-NorPD	PHC services (1) GP psychological consultations (2) EPC psychological consultation	Country of birth (1) Natives (born in Norway with both parents born in Norway) (2) Immigrants (born abroad with both parents from abroad) staying at least 6 months	Age & gender GP and EPC non-psychological consultation	Marital status Income level Length of stay Reason for migration Place of residence	Overall, immigrants are less likely to use a GP or EPC services for mental health problems Immigrant women are somewhat underrepresented in PHC care services for mental health problems
Straiton ML et al. [20] 2016	Norway	2008	1,283,437 individuals Natives (*n =* 1,230,175) Immigrants (*n =* 53,262) 20–67 years	(1) To identify in which forms of treatment immigrant women are over or under represented compared with native Norwegians, and if this varied by country of origin (2) To determine whether use of an interpreter increases the likelihood of accessing different treatment types	Register-base study National registries (1) National Population Register (2) Norwegian Health Economics Administration database-HELFO (3) Norwegian Prescription Database-NorPD	Mental health services (1) Conversational therapy (2) Psychiatric referrals (3) Psychotropic medication (4) Certificates for sickness leave and disability applications	Country of birth (1) Natives (born in Norway with both parents born in Norway) (2) Immigrants (born abroad with both parents from abroad) staying at least 6 months, divided according to the World Bank income categories of their country of origin	Age Diagnosis Use of interpreter	Marital status Income level Length of stay Place of residence	Women are somewhat underrepresented in PHC services for mental health problems A higher percentage of Norwegian women had had a Psychiatric consultation than any of the 6 immigrant groups Psychiatric referral rates did not differ by country of origin
Tarraf W et al. [30] 2014	USA	2000–2008	167,889 individuals US-born (*n =* 133,102) Naturalized FB-citizens (*n =* 14,338) Non-citizens (*n =* 20,449) ≥18 years	(1) Provide a detailed accounting of ED use with policy-relevant immigrant classifications (2) Examine associations between ED use and citizenship status using a Behavioural Model of healthcare access and utilization (3) Determine the most important factors associated with differences in immigrants’ ED services use	Survey-based study (1) Medical Expenditures Panel Survey (MEPS) (2) National Health Interview Survey	Self-reported past-year ED use	Immigration status and place of birth (1) US-born citizens (2) Naturalized foreign-born (FB) citizens (immigrants who have obtained US citizenship) (3) FB non-citizens (legal permanent residents, as well as undocumented and “other” immigrants)	Age & gender Self-reported ethnicity/race Self-rated health Medical conditions Past-year healthcare provider visits Past-year hospital discharges	Insurance status Usual source of care availability Education level Household income-to-poverty Place of residence (urbanity) Region	Immigrants, and particularly non-citizens, were less likely to use ED services Non-citizens are less likely to use ED services and showed that they are also less likely to be repeat users
Tormo MJ et al. [31] 2015	Spain (Murcia)	2006–2008	2453 individuals Natives (*n =* 1303) Immigrants (*n =* 1303) 18–64 years	To describe the utilization of health services among immigrant and male and female native populations	Survey-based study (1) Spanish National Health Survey (SNHS) (2) Health and Culture Survey (SyC)	(1) Unmet healthcare need in the last 12 months (2) Visit to a GP in the last year (3) Visit to dentist in the last year (4) Hospitalization and ED visit in the past year (5) Drug consumption it last 2 weeks	Immigrants with Health Insurance Card (Tarjeta Sanitaria Individual-TSI)	Age & gender Self-assessed health status Health problems last year Activity limitation last 2 weeks	Education level Social class	Migrants showed a lower use of PHC services specialists, but a higher use of ED
Verhagen I et al. [32] 2014	Netherlands	2010	68,214 individuals Natives (*n =* 33,725) Immigrants (*n =* 34,489) ≥55 years	To study whether healthcare use of the four ethnic minority elderly populations in the Netherlands varies from the ethnic Dutch elderly	Register-base study Registry data from the Achmea Health Insurance Company (Achmea)	(1) GP services (2) Receipt of prescriptions (3) Physical therapy (4) Hospital services (5) Medical aids to help with a limitation	Country of birth or surname Turkish, Moroccan, Surinamese and Moluccan	Age & gender	Additional health insurance Neighbourhood deprived	The use of PHC facilities (GP services and prescriptions) within most ethnic minority groups is higher; however, they generally make less use of hospital care, medical aids, and physical therapy
Villarroel N et al. [46] 2015	Spain	2006	22,224 patients Natives (*n =* 20,226) Immigrants (*n =* 1998) 16–64 years	(1) To analyse differences in patterns of healthcare use (visits to PC, hospitalizations and emergency visits) between the native Spanish population and immigrants from the seven leading countries in terms of number of immigrants in Spain in 2006 (2) To examine whether the differences are explained by self-perceived health status, educational level, family characteristics, employment status and social support (3) To determine whether the patterns of association differ by gender	Survey-based study Spanish National Health Survey (SNHS) 2006–2007	(1) Visit to a GP in the 4 weeks before (2) Hospitalization in the past year (3) ED visits in the past year	Country of birth	Age & gender Self-perceived health status	Marital status Educational level Employment status Social support (adapted from the Duke-UNC Functional Social Support Questionnaire) Social support (adapted from the Duke-UNC Functional Social Support Questionnaire)	Immigrants made less than, or about the same use of healthcare services Among men, a lower use of healthcare services was found among those born in Romania for all healthcare levels and among Ecuadorians for hospitalizations Among women a lower use of PHC was found among those born in Argentina, Bolivia and Ecuador, and a higher use among Peruvians. No differences were observed with native-born subjects A higher utilization of healthcare services was only found among men born in Bolivia, who were more likely to use hospitalization
Wang L [47] 2014	Canada	2005–2010	94,948 individuals Canadian-born (*n =* 73,806) Foreign born (*n =* 21,142) 18–75 years	Explore the relationships among individual socio-economic status, residential neighbourhood characteristics and self-reported health for multiple immigrant groups	Survey-based study Canadian Community Health Survey (CCHS)	(1) Have a regular physician (2) Stay overnight in hospital (3) Number of dental visits per year (4) Number of physician visits per year	Country of birth, ethnic origin and immigrant status (1) Native born (2) Long-standing groups (Italian and Portuguese) (3) Recent groups (Chinese and South Asian) (4) Overall foreign born	Age & gender Self-perceived health status Chronic diseases Health behaviour (smoke, overweight, physical activity, vegetable intake)	Marital status Education level Household income Language proficiency Length of stay Neighbourhood characteristics (deprivation & ethnic concentration)	Immigrants have lower rates of overnight stay in hospital All four selected immigrant groups have higher rates for having a regular physician Immigrants report significantly more physician visits Foreign-born groups report fewer dental visits
Wang L et al. [48] 2015	Canada	2005–2010	161,981 individuals Native born (*n =* 124,946) Korean immigrants (*n =* 351) Overall foreign born (*n =* 36,684) ≥25 years	To explore healthcare-seeking behaviour of South Korean immigrants in Toronto, Canada, and how transnationalism shapes post-migration health and health-management strategies	Survey-based study Canadian Community Health Survey (CCHS) 2005–2010	(1) Stay overnight in hospital (2) Physician visits (3) Dental visits	Country of birth (1) Native born in Canada (2) Overall foreign born (3) Korean immigrant	Age & gender Self-perceived health status Chronic diseases	Marital status Education level Employment status Household income Immigration category Length of stay Place of residence	Of the three groups, Koreans use health services the least They have the lowest rate of having a regular doctor and overnight stay in hospital, the lowest numbers for dental and physician visits in the past 12 months, and the highest rate of no doctor visit in the past 12 months

*CMHC* Community Mental Health Centers, *ED* emergency department, *EPC* emergency primary care, *GP* general practitioner, *HIC* high income country, *LIC* low income country, *MIC* medium income country, *OHIP* Ontario Health Insurance Plan, *PHC* primary health care, *STMI* ST segment elevation myocardial infarction

**Fig. 2:**
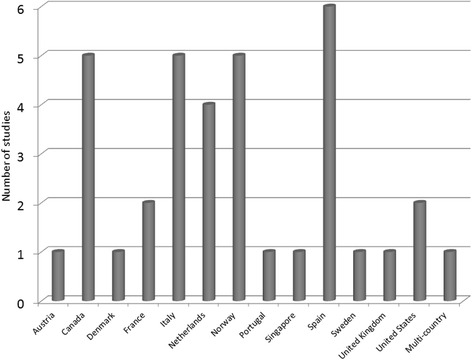
Distribution of studies according to country of destination

Distribution of studies regarding publication year was as follows: 8 studies published in 2013 [17, 22–24, 27, 28, 41, 42], 15 in 2014 [14–16, 19, 21, 30, 32, 33, 35, 36, 38, 40, 43, 44, 47], 10 in 2015 [13, 18, 25, 26, 29, 31, 34, 45, 46, 48] and 3 in 2016 [20, 37, 39]. The majority of the publications analysed data from European countries (28; 78%), both North and Central (12) (Norway [13–15, 19, 20], Denmark [45], Sweden [35], the Netherlands [17, 32, 34, 40] and Austria [41]) and South Europe (15) (France [22, 36], Italy [18, 24, 29, 37, 43], Spain [23, 27, 28, 31, 38, 39, 46] and Portugal [33]) and 1 from the UK [26]. Seven papers (19%) explored this issue in North America (2 from USA [30, 34] and 5 from Canada [16, 21, 25, 47, 48]); and 1 (3%) in Asia (Singapore) [42] (see Fig. 2).

Geographical coverage of the studies has some variation: 21 performed at the national level [13–15, 17, 19–22, 28, 30, 32, 34–36, 38, 40, 41, 45–48], 10 at a regional level [16, 18, 23, 25–27, 29, 31, 37, 44], 3 at a local level [28, 33, 42] and 1 multi-country study [39] with data from a regional level of 1 country and the national level of the other. There were only 4 longitudinal studies (2 prospective [18, 42] and 2 retrospective [27, 43]) and 1 case-control study [35]. Sample sizes ranged from 74 [35] to 7,856,348 [43]. Multivariable regression (Poisson or logistic) was the most frequent analysis. Only 9 studies conducted univariate analysis [29, 32, 33, 35, 38, 43, 48].

### Sources of information

Service utilization could be assessed from two perspectives: the physician’s perspective, based on recorded databases and volume of medical services, and the patient’s perspective, based on patient-reported use of services through healthcare surveys [49].

The largest number of papers (18) used information from administrative [13–16, 18–20, 23, 25, 29, 33, 35, 37, 39, 43] or insurance system databases [32, 34] and specific hospital registries [28] as source of information. Among the 16 papers (44.4%) that analysed healthcare surveys, where people report their individual healthcare use, 14 studies used population-based surveys which were elaborated for other purposes [17, 21, 22, 24, 26, 30, 36, 38, 40, 44, 46–48] while 3 of the surveys were specifically designed to explore immigrants healthcare use [31, 41, 42]. Only 2 studies [33, 45] (5.6%) combined health survey and administrative information and 1 study also used a national survey for general practitioners (GPs) [17].

### Subjects

There were diverse definitions of immigrants. Country of birth was the most common criteria used to define immigrants (18), or country of birth of the subject and their parents (10). In addition, name recognition (2) [32, 34], citizenship (3) [18, 24, 28] or a combination of citizenship and country of birth (3) [30, 42, 45] were also used.

The majority of papers classified the immigrant population in sub-groups usually based on country of birth (13). However, some studies considered geographic area of origin (8) or World Bank categories of income level (5). Other less frequent categories considered were legal status (3), reason of migration (1), length of stay in the country (3) and being first of second generation (1). Only 2 studies (5.6%) [18, 22] compared the use of services considering the immigrant populations as a whole, without defining specific sub-groups in those populations.

### Findings

The outcome “healthcare service utilization” could be organized in seven focus areas: primary care, specialist’s services, hospitalizations, emergency services, mental health, dental care and medication prescription. Some studies reported on more than one outcome. In total, 8 papers analysed the use of primary care (including GP visits, dental care and physiotherapy) [13–15, 21, 27, 36, 44, 48], 6 evaluated the use of specialist services (including hospitalizations or emergency care) [23, 28, 30, 33, 35, 42], 5 assessed mental health services [17, 18, 20, 29, 45], 10 evaluated the use of both primary care and specialists [22, 24, 31, 32, 34, 37, 38, 43, 46, 47], 2 evaluated primary care and mental health [19, 40], 4 evaluated both primary care, mental health and hospitalizations [16, 25, 26, 41] and 1 studied pharmaceutical use and prescriptions [39]. In addition, 6 studies also reported medication consumption [20, 31, 32, 37, 42, 43].

The measurement of healthcare utilization was either continuous (number of contacts) or dichotomic (having had any contact). The period of time used to determine utilization ranged from 4 weeks through 1 year.

The more frequent outcome was that immigrants have lower [17–20, 22, 25, 27, 28, 30, 33, 35, 40, 43, 44, 48] or similar [13, 21, 34, 36, 41, 42] healthcare utilization. However, studies that included analysis by sub-groups of immigrants identified some differences across groups [14–16, 23, 26, 31, 37, 39, 40, 45, 46] as well as with the type of service assessed [14, 24, 29, 31, 32, 38, 40, 46, 47].

The immigrant population showed a similar [23, 24, 29, 31, 32, 34, 36–40, 46] or lower [17, 18, 22, 27, 28, 33, 43] use of primary care and specialized care in countries with universal access to health care—even for undocumented migrants [50]. This finding was consistent regardless of the source of information used. In other countries, some differences were identified associated with the source of information: immigrants showed higher use of health services when estimates were based on surveys [26, 41, 45], while their rates were lower [19, 20, 35] or similar [13–15] when registries or administrative data were used.

## Discussion

The main result of this review is that migrant populations appear to have a lower use of health services than native populations, with a similar level of use of primary care services. This result appears to be independent from differences in need of access. Nevertheless, the great heterogeneity of the studies included in this review, considering both the sources of information, as well as factors used for controlling health need and to classify immigrants in sub-groups, requires caution when making an overall estimation valid for all immigrants.

Different sources of heterogeneity should be mentioned. First, and probably the factor with the highest relevance, was the definition of immigrant and their characterization. This review has identified several factors that could be involved with differences in healthcare utilization among immigrants: income of the original native countries [13–15, 28, 38], the specific reasons motivating migration [15, 16, 19, 25, 26], fluency in the host country language [16, 17, 21, 25, 44, 45, 47] and length of time of stay [13, 15, 19–21, 26, 38, 45, 47, 48].

There were also differences in how medical need was determined and how to estimate factors that predispose to healthcare use. The majority of studies assessed health needs from the point of view of self-perceived health, and through commonly used socio-demographic variables, such as education, income or working status, following the model of Aday and Anderson [51, 52]. Multivariable models were adjusted by these variables to eliminate the effect they could have on utilization, but whether they had a differential influence on immigrants or native populations remains inconclusive.

Variables which could have a significant effect on healthcare service use and in particular for mental health care [53], such as health beliefs and cultural concepts on the part of the immigrants, fear of stigmatization, taboos, perceived efficacy of health interventions or use of alternative services, were usually not considered. The effect of these variables is most commonly explored through qualitative techniques, and papers that used those methods were not included in this report.

Variation in countries’ healthcare systems limits direct cross-country comparisons, although immigrants showed similar patterns of utilization in countries with significant differences in their healthcare services. Nevertheless, studies reviewed pay little attention to the structural and organizational dimensions of healthcare systems, other than reporting the specific conditions for accessing health services. One paper explored the influence of attitudes of professionals regarding immigrants [54], 2 studies assessed the reasons for unmet healthcare need [31, 38] while 2 underscored the patient workload of healthcare professionals [22, 23]. In addition, the effect that new legislation enacted in different countries could have had on access to healthcare services by immigrants has not yet been evaluated and published and therefore cannot be assessed in this review.

Attempting to expanding the scope of previous reviews, we tried not to constrain the inclusion criteria regarding areas of healthcare services assessed [10, 55, 56], context of the study (country) [54, 55], or characteristics of immigrants [54, 55].

This work adds also new information regarding the use of mental health services, both in terms of primary [19, 26] and specialized mental services [16–18, 20, 25, 29, 41, 45]. Nevertheless, and although immigrants have shown a higher susceptibility to emotional and mental health problems that could be linked to the stressors of adapting to the host country [57], those studies reported similar findings as for other health services: an overall lower use by migrants, also with differences across sub-groups and with an occasional higher use of emergency care.

This review also provides the opportunity to have an insight of the healthcare use of certain vulnerable sub-groups, as the handicapped [13], the elderly [13, 15, 32, 37] or patients with chronic conditions [21, 34, 36], but the pattern of use of those sub-groups is similar to that of the general population, even when immigrants seem to have less health problems than natives [13, 34], or a poorer health status [36]. Immigrants also showed a higher use associated with longer periods of stay in the host countries [15, 21] as well as significant differences of use among migrant sub-groups [32, 37].

The effect of gender differences was assessed most notably in papers evaluating the use of mental health services [16, 19, 20, 25, 41, 45]. Nevertheless, no conclusive evidence could be established: compared to their native counterparts, Straiton et al [19, 20] and Durbin et al [16, 25] found a lower use of mental health services for immigrant women, while Kerkenaar et al [41] and Smith-Nielsen et al. [45] found a higher use.

The possibility to analyse the use of different levels of care may help to determine the existence of gaps in utilization (less use in one area could explain an increased use in another area) or highlight the existence of different referral criteria (primary care specialists) [23]. De Luca et al. found [24] an over-utilization of emergency services associated with an under-utilization of preventive care services among the immigrant population. Tormo et al. [31] and Díaz et al. [14] obtained similar results, although they concluded that the higher use of emergency services did not compensate the lower use of GPs. The identification of differences in pharmaceutical consumption could also lead to identify particular health problems or economic barriers accentuated by the development of restrictive health policies.

Lastly, the large number of European studies, particularly from western and central Europe, has to be highlighted, probably depicting the interest about the migratory pressure these countries have faced in the last years—migration from Eastern Europe after the fall of the Iron Curtain; from Latin America, North and sub-Saharan Africa; from internal migration flows south-north after the economic crisis; or most recently, the refugee crisis.

### Study limitations

The literature search was conducted only in one database (MEDLINE), although the electronic search was manually completed using Google Scholar. There were implied limitations in the manual search, since it was not systematized and was susceptible to errors as it relied on title appropriateness (particularly for articles with ambiguous titles). Furthermore, no backward citation of the papers included in the systematic review was performed. Additionally, the systematic search only identified 50% of the papers accepted for inclusion, which raises some doubts regarding the intrinsic limitations of the system to classify and assign terms to papers that compare the use of healthcare services between native and migrants.

Finally, qualitative papers that explored the use of healthcare services were not included, as it would be difficult to draw comparisons from these studies.

## Conclusions

Overall, and regardless of the changes in the immigration process, data here analysed is coincident with results obtained in previous reviews [10, 54, 56], confirming that immigrants show a general tendency to a lower use of health services than native populations. But these data also indicate the existence of differences within the immigrant populations, reinforcing the conclusion that further studies intended to compare the rate of healthcare use between native and immigrant populations should incorporate information that allows for better identification and characterization of the immigrant population. The immigrant population cannot be considered as a uniform whole. Their diversity has to be taking into account when describing and analysing their healthcare utilization. This will also require improvement and standardization of the information collected [55, 58].

In this sense, the limitations of health surveys have to be emphasized. Surveys are not just subjected to memory bias, but they are less suited to be representative of all relevant sub-groups of the immigrant population, as their samples usually do not include enough participants to reflect the wide variability of the diverse immigrant population to estimate their differential use. For instance, only one paper includes immigrants in irregular status [44]. Therefore, the use of data that overcome these limitations has to be encouraged. Further studies should be based on other information, such as registers, administrative or insurance data, or data from non-governmental organizations [59].

## Abbreviations

CMHC: Community Mental Health Centers
ED: Emergency department
EPC: Emergency primary care
GP: General practitioner
HIC: High income country
LIC: Low income country
MIC: Medium income country
OHIP: Ontario Health Insurance Plan
PHC: Primary health care
STMI: ST segment elevation myocardial infarction
